# Neuroinflammation Induced by Intracerebroventricular Injection of Microbial Neuraminidase

**DOI:** 10.3389/fmed.2015.00014

**Published:** 2015-03-17

**Authors:** Pablo Granados-Durán, María D. López-Ávalos, Jesús M. Grondona, María del Carmen Gómez-Roldán, Manuel Cifuentes, Margarita Pérez-Martín, Martina Alvarez, Fernando Rodríguez de Fonseca, Pedro Fernández-Llebrez

**Affiliations:** ^1^Departamento de Biología Celular, Genética y Fisiología, Instituto de Investigación Biomédica de Málaga (IBIMA), Facultad de Ciencias, Universidad de Málaga, Málaga, Spain; ^2^Centro de Investigaciones Biomédicas en Red de Bioingeniería, Biomateriales y Nanomedicina, Facultad de Ciencias, Universidad de Málaga, Málaga, Spain; ^3^Departamento de Anatomía Patológica, Facultad de Medicina, Universidad de Málaga, Málaga, Spain; ^4^Unidad de Gestión Clínica de Salud Mental, Hospital Regional Universitario de Málaga, Instituto de Investigación Biomédica de Málaga (IBIMA), Málaga, Spain

**Keywords:** inflammation, brain, ventricle, neuraminidase, ependyma

## Abstract

In the present paper, we describe the facts that took place in the rat brain after a single injection of the enzyme neuraminidase from *Clostridium perfringens* into the right lateral ventricle. After injection, it diffused through the cerebrospinal fluid of the ipsilateral ventricle and the third ventricle, and about 400 μm into the periventricular brain parenchyma. The expression of ICAM1 in the endothelial cells of the periventricular vessels, IBA1 in microglia, and GFAP in astrocytes notably increased in the regions reached by the injected neuraminidase. The subependymal microglia and the ventricular macrophages begun to express IL1β and some appeared to cross the ependymal layer. After about 4 h of the injection, leukocytes migrated from large venules of the affected choroid plexus, the meninges and the local subependyma, and infiltrated the brain. The invading cells arrived orderly: first neutrophils, then macrophage-monocytes, and last CD8α-positive T-lymphocytes and B-lymphocytes. Leukocytes in the ventricles and the perivascular zones penetrated the brain parenchyma passing through the ependyma and the glia limitans. Thus, it is likely that a great part of the damage produced by microorganism invading the brain may be due to their neuraminidase content.

## Introduction

In agreement to Graeber et al. (1) the term neuroinflammation implies not only microglial activation but also the invasion of the nervous tissue and/or the cerebrospinal fluid (CSF) by blood-circulating leukocytes. Inflammation of the brain can be triggered by the presence in the ventricle, the meninges, or the nervous parenchyma of biological agents such as bacteria or viruses, or by a plethora of chemical or mechanical stimuli (2–5). In any case, the clinical relevance of brain inflammation lies in part on the detrimental or even lethal effects of the invading defensive cells or proteins, which eventually can give rise to chronic neurodegenerative diseases (6, 7).

Among the many agents that trigger neuroinflammation some are located in the microbial envelope, such as lipopolysaccharides (8, 9) or the enzyme neuraminidase (NA) (10). Indeed, the abundance of NA in the outer surface of certain viruses and bacteria has been related to their pathogenicity (2, 11–14). So much so that a single mutation in the hemagglutinin-NA (HN) surface complex of the mump virus is enough as to greatly decrease its pathogenicity, an interesting feature in the design of vaccines (15). Moreover, inhibitors of the NA activity are among the most important antiviral compounds [i.e., oseltamivir; (16)]. Despite all evidences supporting a pre-eminent role of NA in neuroinflammation, little is known about its exact mechanism of action and the events that are triggered after its administration. Moreover, the molecular differences among the neuraminidases that can be found in the microbial covers, probably lead to differences in the pathogenesis that they provoke (17).

*Clostridium perfringens* is a ubiquitous bacterium that possesses NA at its surface (18). NA from *C. perfringens* is easily available, so we used it before to desialylate some molecules present in the ventricular CSF (19). We reported that a single intracerebroventricular (ICV) injection of a high dose of NA (about 10 μg) produced the massive loss of the multiciliated ependyma followed by acute inflammation of meninges and ventricles and the development of hydrocephalus (10). By decreasing the amount of injected NA, only a partial detachment of the ependymal layer occurred in the injected lateral ventricle, but not in the contralateral ventricle or the third ventricle. This model turned also useful to investigate the importance of the ependyma on postnatal neurogenesis (20). We investigated the time course of inflammatory events after ICV administration of low doses of NA from *C. perfringens*, what might prove to be a useful model to study neuroinflammation. The facts suggest the importance of NA in degenerative events known to occur after microbial invasion of the central nervous system.

## Materials and Methods

### Animals

Eighty-four adult male Wistar rats (body weight, bw, 250–300 g) were used in this study (Charles River Laboratories, Barcelona, Spain). All animals were housed under a 12 h light/dark cycle with food and water available *ad libitum*. Animal procedures were done according to the European Union (86/609/EEC) and Spanish (RD 1201/2005) legislations.

### Intracerebroventricular injection

Animals were anesthetized with 2,2,2-tribromoethanol (0.2 g/kg body weight, Fluka Chemika, St. Louis, MO, USA) and positioned in a stereotaxic frame. NA from *C. perfringens* (Roche Diagnostics, Basel, Switzerland, catalog No. 11 585 886 001) dissolved in 0.9% sterile saline (25 mU/μl) was administrated by stereotaxic surgery into the right lateral cerebral ventricle, in the following coordinates from Bregma: antero-posterior = −0.5 mm; medio-lateral = −1.4 mm; dorso-ventral = −3.5 mm (21). With the aid of a pump, a 500 mU (20 μl) of NA were injected at a rate of 2 μl/min during 10 min. Control rats were injected with saline. Some animals were sacrificed immediately after NA injection (time 0 h), and the rest were recovered from anesthesia and sacrificed at 2, 4, 12, 24 h, 2, 4, 7, 15, 30 days, 3, and 6 months after the administration of NA.

### Histological procedures

Animals were anesthetized with 2,2,2-tribromoethanol and transcardially perfused with saline followed by Bouin’s fixative. Brains were removed and immersed in the same fixative for 24 h at room temperature (RT), and later embedded in paraffin wax. Seven-micrometer sections were obtained from each brain, and series along the region of interest (from Bregma −0.3 to −1.2 mm approximately) were mounted in gelatine treated slides.

### Lectin binding

The *Limax flavus* agglutinin (LFA, Calbiochem, San Diego, CA, USA) was chosen for its exclusive affinity for sialic acid. Deparaffinized thin sections were incubated with 4 μg/ml LFA in lectin buffer (1 mM Cl_2_Ca, 1 mM Cl_2_Mg, 0.6% TRIS, pH 7.6) for 1 h at RT. The lectin bound to the tissue was later identified using a rabbit anti-LFA antibody [(22); see Immunohistochemistry]. The peanut (*Arachis hypogea*) agglutinin (PNA, Vector, Burlingame, CA, USA. l-1070) has affinity for terminal residues of galactose, which is the subterminal sugar residue in complex-type glycoproteins. After the removal of terminal sialic acid, galactose is exposed as terminal residue, and the PNA binding sites arise. To check for the removal of sialic acid by the action of NA, sections were incubated in 3 μg/ml peroxidase-labeled PNA in 0.1 M phosphate buffered saline (PBS) for 1 h at RT. The peroxidase activity was detected with 0.1 M PBS containing 0.05% diaminobenzidine (DAB; Sigma) and 0.03% hydrogen peroxide (Merck).

### Immunohistochemistry

Immunohistochemistry was carried out on deparaffinized tissue sections using either immunoperoxidase or immunofluorescence techniques. The primary and secondary antibodies used are listed in Tables 1 and 2, respectively. All of them were diluted with PBT buffer (0.3% bovine serum albumin, 0.3% Triton X-100 in PBS pH 7.3). Primary antibodies were incubated overnight at RT. Negative controls for the immunostaining consisted in equivalent sections subjected to the same protocol but omitting the primary antibody.

**Table 1:** **List of primary antibodies used for immunohistochemistry**.

Antibody	Inmunogen, expected target	Manufacturer, species and type, catalog number	Dilution
Anti-CD3ε	C-terminus of mouse CD3ε, T-lymphocytes	Santa Cruz, Dallas, TX, USA, goat IgG polyclonal, SC1127	1:200
Anti-CD8α	Rat thymocyte membrane CD8α glycoprotein, CD8+ T-lymphocytes	Serotec, Oxford, UK, mouse IgG monoclonal, MCA48R	1:1500
Anti-GFAP	GFAP from pig spinal cord, astrocytes	Sigma, St Louis, MO, USA, mouse IgG monoclonal, G3893	1:1000
Anti-IBA1	Carboxy-terminal sequence of IBA1, microglia	Wako. Osaka, Japan rabbit IgG polyclonal,19-19741	1:500
Anti-ICAM1	Rat ICAM1 CD54, vascular endothelial cells	R&D Systems, Minneapolis, MN, USA, goat IgG polyclonal, AF583	1:40
Anti-IL1β	*E. coli-*derived recombinant rat interleukin1β, macrophagues	R&D Systems, Minneapolis, MN, USA, goat IgG polyclonal, AF501NA	1:200
Anti-LFA	Agglutinin of the lectin *Limax flavus*, LFA	A gift of Prof. E Rodríguez, rabbit IgG polyclonal	1:1000
Anti-MPO	Myeloperoxidase from human granulocytes, neutrophils	Abcam, Cambridge, UK, rabbit IgG polyclonal, ab9535	1:100
Anti-PAX5	C-terminus of human Pax-5 B-lymphocytes	Santa Cruz, Dallas, TX, USA, goat IgG polyclonal, SC1974	1:200

**Table 2:** **List of secondary antibodies used for immunohistochemistry**.

Antibody	Supplier, cat. number	Dilution
**Immunoperoxidase**
Goat anti-mouse IgG (biotinilated)	Pierce, Rockford, IL, USA, 31800	1:1000
Goat anti-rabbit IgG (biotinilated)	Pierce, Rockford, IL, USA, 31820	1:1000
Horse anti-goat IgG (biotinilated)	Vector, Burlingame, CA, USA, VA9500	1:1000
**Immunofluorescence**
Rabbit anti-goat IgG (Alexa 488)	Molecular Probes, Madrid, Spain, A21222	1:1000
Goat anti-rabbit IgG (Alexa 488)	Molecular Probes, Madrid, Spain, A11070	1:1000
Donkey anti-mouse IgG (Alexa 594)	Molecular Probes, Madrid, Spain, A21203	1:1000
Donkey anti-rabbit IgG (Alexa 594)	Invitrogen, Carlsbad, CA, USA, A21207	1:1000
Donkey anti-goat IgG (Alexa 488)	Invitrogen, Carlsbad, CA, USA, A11055	1:1000

Sections for immunoperoxidase procedures were incubated in 3% hydrogen peroxide and 10% methanol in PB 0.1 M to quench endogenous peroxidase activity, prior to overnight incubation with the primary antibody. Biotinylated secondary antibodies were used (Table 2; 60 min incubation), along with the avidin-biotin-complex (ABC) amplification method. The ABC reagent was prepared according to manufacturer’s instructions (Thermo Fisher Scientific, Waltham, MA, USA) and incubated during 30 min. Peroxidase activity was revealed with 0.05% DAB and 0.03% hydrogen peroxide. All the incubations were performed in a moist chamber at RT.

To study the co-localization of particular markers, double immunofluorescence was performed using the following pairs of antibodies: ICAM1 and IBA1, IBA1, and IL1β, myeloperoxidase (MPO) and IL1β, and CD3ε and CD8α. The primary antibodies were mixed and incubated simultaneously (overnight at RT). Alexa fluor secondary antibodies (Table 2) were used. Coverslides were mounted with buffered glycerol containing 10% of the anti-fading agent Mowiol 4-88 (Calbiochem/EMD Chemicals, San Diego, CA, USA). The sections were then observed under a confocal microscope (Leica, SP5 II). Some antibodies required specific antigen retrieval pretreatment prior to immunostaining. Thus, the sections incubated with anti-CD3ε and anti-CD8α were pretreated with 10 mM TRIS-EDTA buffer pH 9.0, and the sections incubated with anti-PAX5 were pretreated with 10 mM citrate buffer pH 6.0. In both cases, pretreatment was done at 95°C in microwave for 8 months.

### Quantitative studies

Two different strategies were used depending on the staining properties of the cells or structures to be quantified. When the quantified resident cells displayed processes or irregular shapes (microglia, astrocytes, and endothelial cells) the immunostained area was measured. If the cells had well-defined profiles easily distinguishable from each other (infiltrating leukocytes), a direct count of the number of cells was preferred.

#### Quantification of ICAM1, IBA1, and GFAP immunostaining

To study the response of the resident cells to NA-injection, we quantified the area immunostained with ICAM1, IBA1, and GFAP at different times (0, 4, 24 h, 7, and 15 days) after the inoculation of NA. These times were determined after the qualitative observation of the differences between the injected and the contralateral sides over time.

A previous detailed study of immunostained serial sections allowed the selection of the levels to be studied. Thus, two sections from each animal (Bregma −0.3 and −0.92 mm approximately) were chosen for the quantification. In any case, the selected sections were close to the foramen of Monro but far enough from the site of injection as to avoid areas of surgically damaged tissue. In each section studied, micrographs were taken from the striatal ventricular wall, and from the septum-fimbria wall, both in the injected side and in the contralateral one (red squares in Figure 2A show the approximate location of the micrographs). Pictures were then processed using the image analysis software Visilog 6.3 (Noesis, France). To introduce a correction related to the basal level of expression of a particular marker in each animal, the immunostained area measured in the contralateral side was subtracted to the value obtained in the same location in the injected side. This correction was made to subtract the basal expression of a marker in each animal, which was found to vary from rat to rat. Afterwards, the mean value was obtained from the two locations and the two sections studied from each animal, which was considered the total stained area of that animal. A total of five animals were analyzed in each time post-injection studied.

#### Quantification of infiltrating leukocytes

Cell infiltration was evaluated by immunostaining with the following antibodies: anti-MPO for neutrophils, anti-IL1β for monocyte-macrophages, anti-CD3ε for T-lymphocytes, anti-CD8α for cytotoxic T-lymphocytes, and anti-PAX5 for B-lymphocytes. We realize that these substances could also be synthesized by other cell types. However, presently and in agreement to many researchers, these markers are used to identify these different kind of immunologic blood-borne cells with a high degree of confidence. However, a low possibility exists that some cell does not belong to the corresponding group.

Immunoreactive cells were counted in three different locations: (1) the choroid plexus within the injected ventricle; (2) the meninges close to the optic chiasm; and (3) the zone around the foramen of Monro containing large venules close to the origin of the choroid plexus and the subfornical organ. Serial sections from Bregma −0.85 to −1.13 mm approximately (the level of the foramen of Monro) were immunostained with each antibody. Four sections per animal were selected for counting of positive cells. Between three and five animals were studied in each time post NA-injection. The times post-injection studied were the same as those selected for ICAM1, IBA1, and GFAP quantification (0, 4, 24 h, 7, and 15 days). In the case of the choroid plexus, the area occupied by this structure in each section studied was measured with the Visilog 6.3 software; the number of positive cells counted in this location was expressed per millimeter square of choroid plexus. In the other two locations, the number of positive cells was expressed per section.

#### Statistical analysis

Differences between times post-injection of NA were evaluated by ANOVA (significance level of 0.05), followed by a Tukey *post hoc* test with a level of significance of 0.05. The values measured at time 0 were considered the baseline. The software SPSS^®^ Statistics 20 (IBM^®^) was used for this analysis. In the histograms, the existence of significant differences between groups is indicated with different letters. Thus, two bars labeled with the same letter means that there is no significant difference between the two groups. Conversely, two bars labeled with different letters indicate that there is a significant difference between them.

## Results

**Figure 1:**
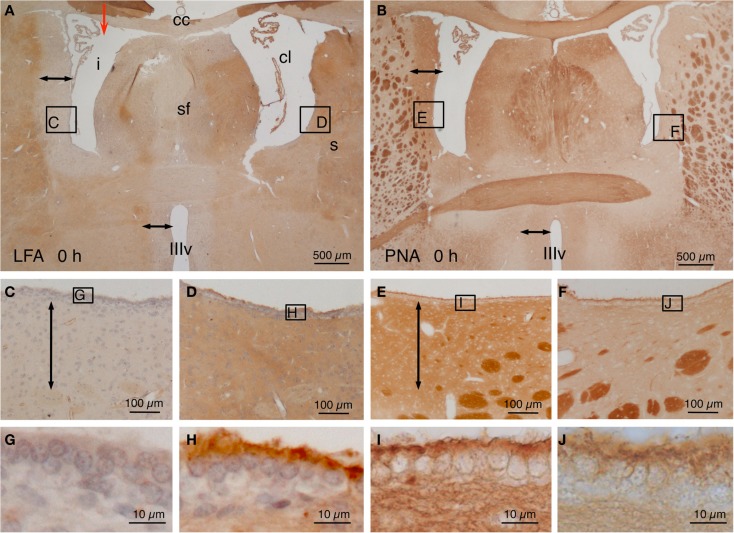
**Lectin histochemistry in NA-injected rat brains immediately after the injection**. **(A,B)** The site of injection is indicated with a red arrow in **(A)**. Note a LFA-negative and PNA-positive halo around the injected lateral ventricle (i) and the third ventricle (IIIv) (double-headed arrow) but not the contralateral ventricle (cl). **(C–F)** Details of the zones squared in **(A,B)** showing the negative or positive halo (double-headed arrows). The squared areas are shown in detail in the following pictures. **(G–J)** The surface of the ependymal cells was LFA-negative **(G)** and PNA-positive **(I)** in the injected ventricle, whereas it was LFA-positive **(H)** and almost PNA-negative **(J)** in the contralateral ventricle. cc, corpus callosum; cl, contralateral ventricle; i, injected lateral ventricle; s, striatum; sf, septum-fimbria; IIIv, third ventricle. Sections in **(A,C,D,G,H)** were counterstained with hematoxilin–eosin.

Animals were sacrificed immediately after the injection of NA (T: 0 h), LFA lectin exhibited a negative halo (~400 μm from the ventricle surface) around the injected lateral ventricle and the rostral third ventricle (Figures 1A,C). The ependymal surface of the injected lateral ventricle showed no staining at all (Figure 1G). Conversely, the contralateral ventricle showed no halo (Figures 1A,D) and a conspicuous staining of the subependyma and the surface of the ependymal cells (Figure 1H). On the contrary, when using PNA lectin (terminal galactose affinity) a positive label was found in the subependyma of the injected ventricle (Figures 1B,E) that was particularly intense in the surface of the ependymal cells (Figure 1I). The walls of the contralateral ventricle showed virtually no binding to PNA (Figures 1B,F), and the surface of the ependymal cells was only feebly positive (Figure 1J). After about a week, the ependymal cells that remained in the ventricular lining of the injected ventricle recovered the sialic acid and thus the LFA binding.

Control rats, which were injected with the vehicle, did never present any sign of brain inflammation. Only the brain cortex at the level of the needle path showed a few signs of local inflammatory reaction that spontaneously healed as observed in brains examined at different times after the injection. No evidences of inflammation were observed in the brain ventricles, the meninges or the periventricular regions.

The elimination of sialic acid triggers a series of events resulting in inflammation. Some responses depend on changes in resident brain cells while others on infiltrating blood leukocytes. Therefore, both cell populations were quantified afterwards.

### NA induces upregulation of IBA1, GFAP, and ICAM1 in resident cells

**Figure 2:**
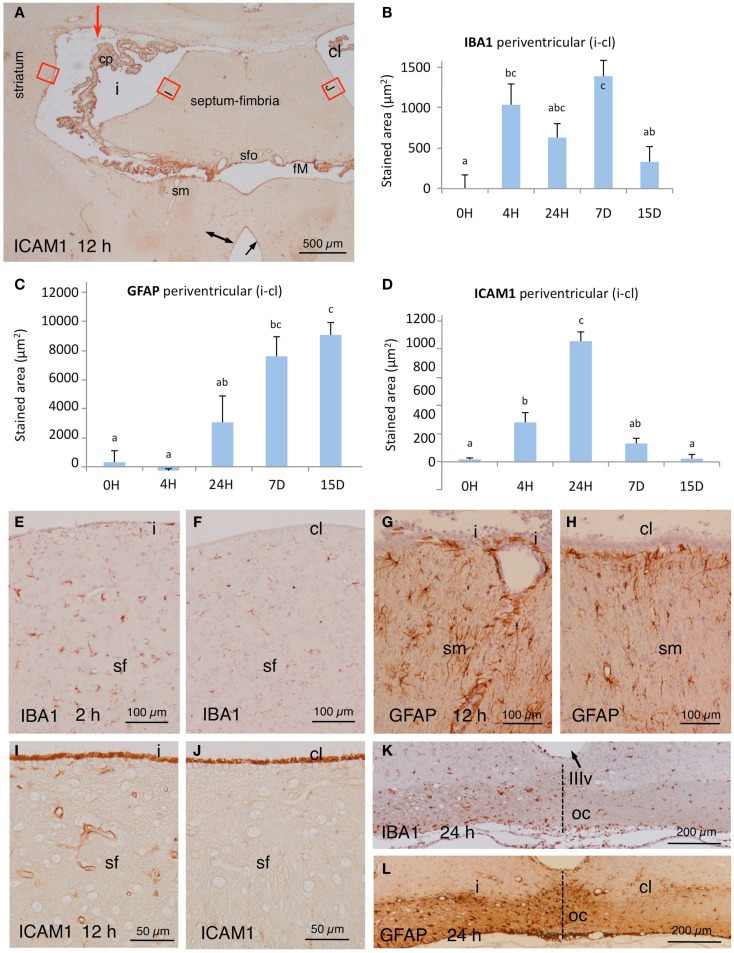
**(A)** Panoramic view at the level of the foramen of Monro (fM) of the brain of an animal sacrificed 12 h after NA injection (red arrow points injection site). The tissue was immunostained with ICAM1. Note an increase in the immunoreactivity in the vessels of the affected periventricular zones (double-headed arrow) and the strong reactivity of the choroid plexus (cp) and the surface of the ependymal cells (arrow). The red squares correspond roughly to the zones used for quantification; some are detailed in **(I,J)**. **(B–D)** Quantification of the expression of IBA1, GFAP, and ICAM1 in the periventricular area at selected times post-injection. Red squares in **(A)** point approximately the areas where micrographs were taken in the striatum and the septum. The values represented are the difference of stained area between the injected lateral ventricle (i) minus de contralateral ventricle (cl). The bars are the mean ± SEM of five animals analyzed. Letters (a–c) on bars indicate the absence (same letter) or presence (different letter) of a significant difference between groups (α = 0.05). **(E,F)** Immunoreactivity to IBA1 in the periventricular region at the level of the septum-fimbria (sf) in the injected (i) and the contralateral (cl) ventricles, 2 h after NA injection. **(G,H)** Details of GFAP staining in the stria medullaris (sm) in the injected (i) and the contralateral (cl) sides 12 h after the administration of NA. **(I,J)** Details [red squares in **(A)**] of the immunoreactivity to ICAM1 in the septum-fimbria (sf) subependymal parenchyma of the injected (i) and the contralateral (cl) sides 12 h after the injection of NA. Note the strong immunoreactivity of the ependymal surface in both ventriculi. **(K,L)** Twenty-four hours after NA injection, the optic chiasm (oc) beneath the third ventricle (IIIv) showed a higher IBA1 and GFAP staining in the injected (i) than in the contralateral side (cl). cl, contralateral ventricle; cp, choroid plexus; fM, foramen of Monro; i, injected lateralventricle; sfo, subfornical organ; sm, stria medularis; sf, septum-fimbria; oc, optic chiasm; IIIv, third ventricle.

In NA-injected animals, the immunoreactivity to IBA1 antiserum increased notably in the affected periventricular zones (compare Figures 2E,F). We quantified the differences in two selected regions of the striatum and the fimbria-fornix at different post-injection times. IBA1 was upregulated from early times after NA injection, and the basal levels were recovered after about 2 weeks (Figure 2B). A notable increase in IBA1 immunoreactivity was also evident in the injected side in places located far from the site of injection, as is the optic tract (Figure 2K).

GFAP staining in astrocytes of control rats (non-injected or saline-injected) was as expected, with no staining detected in the ependyma or the choroid plexus. As for IBA1, quantification of the GFAP stained area was carried out in selected regions of the striatum and the fimbria-fornix area of the injected ventricle relative to the contralateral one. GFAP staining showed a higher expression 24 h after NA-injection, which further increased one week later. It remained overexpressed even 2 weeks post-injection (Figure 2C). The increase in immunoreactivity was clearly observed also in other region such as the stria medullaris (compare Figures 2G,H). Regions located farther from the site of injection also presented increased GFAP expression in the injected side with respect of the contralateral one, a fact particularly evident in the optic tract (Figure 2L).

In control rats, the wall of the large vessels of the meninges, the choroid plexus, the subependyma around the foramen of Monro, and the surface of the ependymal cells (especially of the choroid plexus) were immunoreactive to ICAM1. Parenchymal capillaries were virtually negative. Conversely, in the injected rats the wall of the parenchymal vessels located in the subependyma of the affected ventricles (up to ~400 μm from the ventricle surface) became ICAM1-immunoreactive (Figure 2A; compare Figures 2I,J). As for IBA1 and GFAP, the changes in immunoreactivity to ICAM1 were quantified in the same selected zones of the lateral ventricle wall. There was a progressive increase in immunoreactivity that was evident 4 h after the injection and peaked at 24 h (Figure 2D). ICAM1 expression decreased afterwards to reach control levels after about 2 weeks.

**Figure 3:**
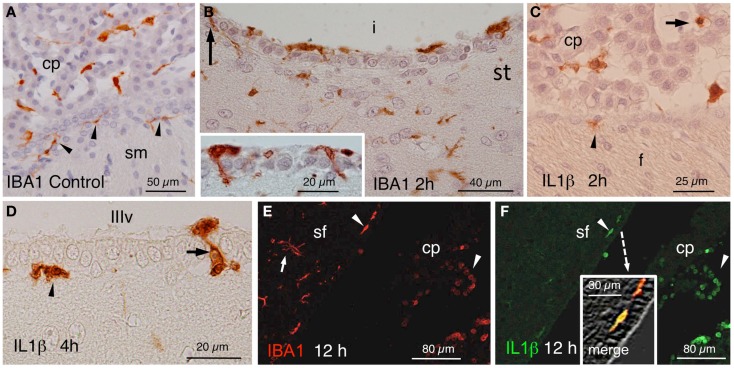
**(A)** IBA1-positive epiplexus and subependymal cells (arrowheads) in a vehicle-injected specimen sacrificed immediately after the injection. **(B)** Detail of the striatal (st) side of the injected lateral ventricle (i) near the inferior sulcus of the lateral ventricle 2 h after the injection. Note IBA1-positive cells over, beneath and among the ependymal cells (arrow and inset). **(C)** Two hours after the injection of NA, scattered IL1β-positive cells occurred inside (arrow) and outside the choroid plexus (cp) as well as in the subependyma (arrowhead) of the injected ventricle. **(D)** Detail of IL1β-stained cells in the subependyma (arrowhead) and crossing the ependymal wall (arrow) of the third ventricle (IIIv) 4 h after the injection of NA. **(E,F)** Double immunofluorescence with anti-IBA1 (red) and anti-IL1β (green). Double-stained cells appear in the ventricle and the subependyma (arrowheads, and merge detailed in the inset of **(F)** showing a double-stained subependymal cell). Many IBA1-positive cells in the brain parenchyma were not immunoreactive to IL1β [arrow in **(E)**]. cp, choroid plexus; sf, septum-fimbria; sm, stria medullaris; (i), injected lateral ventricle; st, striatal wall of the lateral ventricle; f, fornix; IIIv, third ventricle.

In control rats, IBA1 stained epiplexus cells in the choroid plexus and very few subependymal microglial cells (Figure 3A). In the experimental animals, a quite striking observation was that some IBA1-positive cells beneath the ependyma, seemed to penetrate between the ependymal cells even before the onset of blood cell infiltration (Figure 3B and inset). In similar locations, IL1β-positive cells could be found at early times after NA administration (Figures 3C,D). Moreover, double immunostaining demonstrated the existence of cells that were positive to both IBA1 and IL1β (Figures 3E,F).

### NA induced extravasation of blood leukocytes

**Figure 4:**
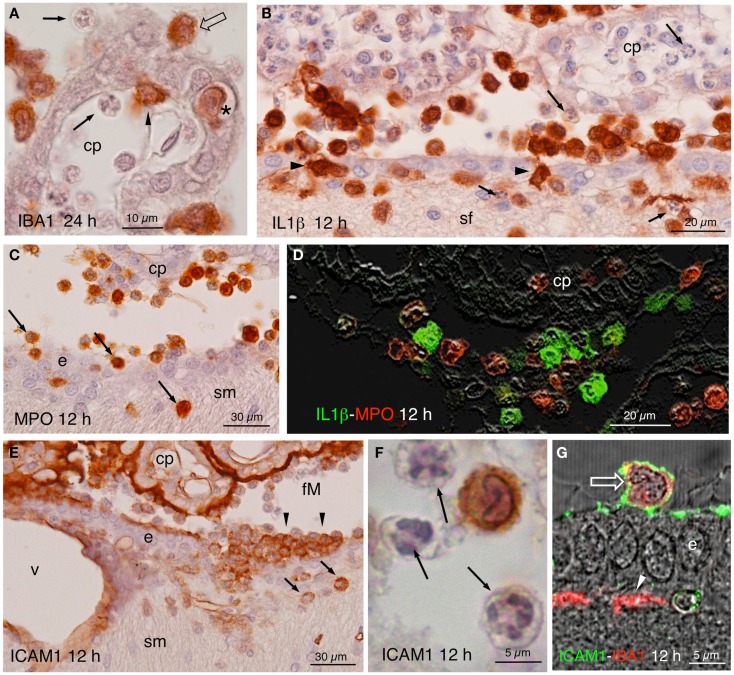
**(A)** Infiltrating IBA1-positive cells in the choroid plexus (cp) of the injected ventricle at 24 h post-injection. Polymorphonucleated cells inside and outside the choroid plexus were never stained (arrows); positive cells occurred inside (arrowhead) outside (open arrow), and in transit (asterisk). **(B)** Twelve hours after NA injection, abundant IL1β-positive cells were seen in the ventricle, penetrating the ependyma (arrowheads) and in the brain parenchyma, in the septum-fimbria (sf) area. Polymorphonuclear negative cells, present inside and outside choroid plexus (cp), the ventricle and the parenchyma (arrows), were also numerous. **(C)** MPO immunostaining in the region of the foramen of Monro, where positive cells were found near the choroid plexus (cp), the ependyma (e), and penetrating the brain parenchyma (arrows) in the region of the stria medullaris (sm). **(D)** IL1β (green) and MPO (red) double immunofluorescence in the region of the choroid plexus (cp) of the injected ventricle 12 h after NA administration. No double-stained cells were detected. **(E)** Detail of a zone of the foramen of Monro (fM) comprising a part of the stria medullaris (sm) 12 h after the injection of NA, immunostained with anti-NCAM1. The surface of the ependymal cells (e) and of the choroid plexus (cp) and the wall of the local vessels (v) was strongly stained. Some infiltrating cells in the ventricle (arrowheads) and in the brain parenchyma (arrows) were also positive. **(F)** Detail of an immunoreactive cell with lobed nucleus in the ventricle. The polymorphonucleated leukocytes (arrows) were never immunoreactive to ICAM1. **(G)** Some cells present in the ventricle were double-stained with ICAM1 and IBA1 (open arrow). The ICAM1-positive surface of the ependymal cells (e) can be observed, as well as some IBA1-positive subependymal cells (arrowhead). cp, choroid plexus; sf, septum-fimbria; e, ependyma; sm, stria medullaris; fM, foramen of Monro; v, vessels.

**Figure 5:**
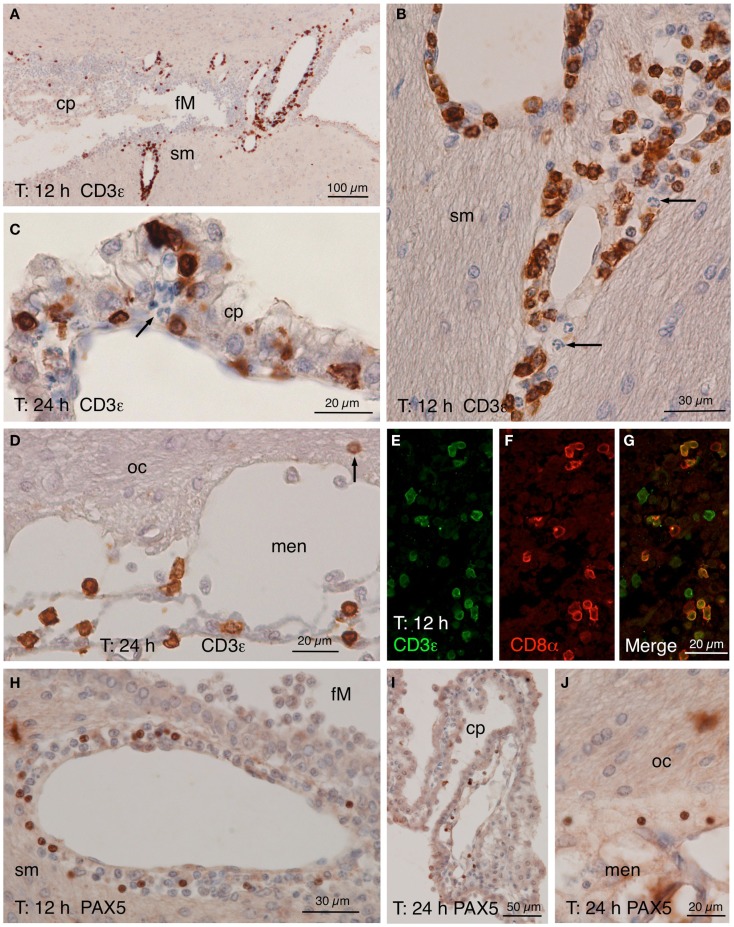
**CD3ε, CD8α, and PAX5 immunostainings**. **(A–D)** CD3ε staining in the zone of the foramen of Monro (fM) of the injected ventricle comprising a part of the stria medullaris [sm, **(A)**]. Many positive cells occurred in the perivascular spaces of the local large venules 12 h after NA injection. A detailed view **(B)** of the vessels in the stria medullaris (sm) shows that many positive cells have smooth nuclei while polymorphonucleated cells (arrow) are negative. **(C)** Twenty-four hours after the injection, some positive cells seemed to cross the ependyma of the choroid plexus (cp). **(D)** Positive cells were also present in the meninges (men) next to the optic chiasm (oc), and few appeared to penetrate the brain parenchyma (arrow). **(E–G)** Double immunofluorescence of lymphocytes in a 12 h post-injection brain section. Most CD3ε-positive cells [**(E)**, green] found in the perivascular spaces of the large vessels were also positive for CD8α [**(F)**, red]. **(H–J)** PAX5 immunostaining showing positive cells in the wall of the large vessels around the foramen of Monro [fM; **(H)**], although they were not as abundant as CD3ε-positive cells. Few PAX5-positive nuclei were also found in the choroid plexus (cp) of the injected lateral ventricle **(I)** as well as in the meninges (men) close to the optic chiasm [oc; **(J)**]. cp, choroid plexus; fM, foramen of Monro; men, meninges; oc, optic chiasm; sm, stria medullaris.

The most noticeable observation after the ICV inoculation of NA was the extravasation of leukocytes in the injected, but not the contralateral side. From about 4 h after the injection, cellular infiltration took place in large blood vessels located in the meninges, choroid plexus, and subependymal parenchyma. Extravasation in smaller vessels was infrequent. The extravasated leukocytes penetrated the brain parenchyma directly from the vessels passing through the blood–brain barrier, the pial barrier, or the ependyma of the choroid plexus (Figure 4A) and of the ventricle walls (Figures 4B,C,E). To identify the nature of infiltrating cells, we used antibodies against MPO for neutrophils (Figures 4C,D), IL1β for monocyte-macrophages (Figures 3F and 4B,D), CD3ε and CD8α for T-lymphocytes (Figures 5A–G), and PAX5 for B-lymphocytes (Figures 5H–J). Some infiltrating cells, seemingly of the macrophagic-monocyte lineage, also stained for IBA1 (Figures 3E and 4A) and ICAM1 (Figures 4E–G). Double immunostaining with anti-MPO and anti-IL1β (Figure 4D) revealed no co-localization of these two markers, indicating that the antibodies discriminate quite well between neutrophils and macrophages. Moreover, no polymorphonucleated cell was stained using anti-IL1β, IBA1 or ICAM1 at any time post-injection (Figures 4A,B,F). It is likely that the monocyte-macrophage cells may be recognized by the IBA1 antiserum. Moreover, some of them (i.e., those that were activated) were also recognized by IL1β and ICAM1 antibodies (Figures 4B,D,E–G).

Lymphocytes infiltrated from the same vessels that neutrophils and macrophages (Figure 5), crossed the choroid plexus ependyma (Figures 5C,I), and invaded the ventricle (Figures 5A,I), the meningeal spaces (Figures 5D,J), and the brain parenchyma (Figures 5B,H). As revealed by double immunofluoresce using anti-CD3ε and anti-CD8α antibodies, most of them were cytotoxic T-lymphocytes (Figures 5E–G). B-lymphocytes (PAX5-positive) were also present, but they were much less abundant (about one-tenth) than T-lymphocytes (Figures 5H–J).

**Figure 6:**
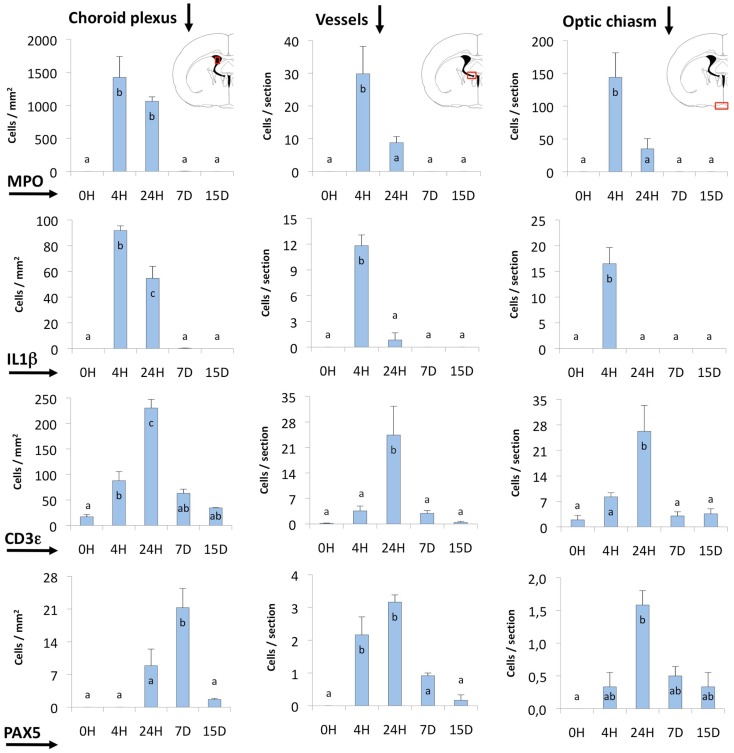
**Quantification of MPO, IL1β, CD3ε, and PAX5 immunostained cells**. The quantification was done in three different locations (indicated by red squares in the coronal section diagrams): (i) the choroid plexus of the injected lateral ventricle, (ii) the area comprising large vessels close to the foramen of Monro and near the subfornical organ (Vessels), and (iii) the zone of the meninges and the optic chiasm. Most of the cells stained and counted were infiltrating cells. MPO- and IL1β-positive cells (neutrophils and macrophagic cells, respectively) reached a maximum before 24 h. The peak of CD3ε and PAX5-positive cells (probably T- and B-lymphocytes) occurred later, and these cells remained longer (even up to 15 days) in some locations. The bars represent the mean ± SEM of five animals. Letters (a–c) on bars indicate the absence (same letter) or presence (different letter) of a significant difference between groups (α = 0.05). Time 0 post-injection of NA (0 h) is considered the baseline population of positive cells.

Infiltrating cells were counted at different post-injection times in three selected locations (Figure 6): (i) the choroid plexus of the injected ventricle (Choroid plexus in Figure 6); the contralateral plexus showed virtually no activation; (ii) the zone around the ipsilateral foramen of Monro, where large venules are found (Vessels in Figure 6); (iii) the region of the meninges close to the ipsilateral side of the optic chiasm (Optic chiasm in Figure 6).

Neutrophils were by far the most numerous infiltrating cells, followed by T-lymphocytes and monocyte-macrophages, while B-lymphocytes were the least abundant. The choroid plexus was the richest source of blood cells, followed by the large vessels in the meninges and those around the foramen of Monro. The meningeal vessels, although situated far from the site of injection (i.e., the optic chiasm) were quite reactive.

Regarding the time course of infiltration, the earliest events were the invasion of neutrophils (MPO-positive; Figures 4C,D) and macrophages (IL1β-positive; Figures 4B,D), which reached a maximum only 4 h after the inoculation of NA; at 24 h these cells were already clearing (Figure 6). Lymphocytes (CD3ε-positive; Figures 5A–D) appeared later, and peaked at about 24 h (Figure 6). In the choroid plexus, these cells were more persistent and remained for up to 2 weeks.

## Discussion

The present work describes a novel model of neuroinflammation based on the ICV administration of the enzyme NA, which specifically removes terminal sialic acid from carbohydrate chains. This model intends to demonstrate that the NA activity from brain-targeting pathogens might result in pleiotropic inflammatory changes. The events that follow NA administration closely resemble those of an inflammatory process, and could be divided into phases. The phase 1 comprises the first 4 h after injection; the events that take place during this period are (a) the cleavage of sialic acid from the ependymal surface and the periventricular parenchyma, (b) the detachment and death of a fraction of the ependymal cells; and (c) the response of resident cells such as ventricular macrophages, microglia, astrocytes and endothelial cells of the vessels. The phase 2 starts about 4 h after NA injection. Extravasation of leukocytes occurs from certain brain vessels, resulting in the invasion of the affected zones in an ordered sequence of neutrophils, macrophages, and lymphocytes. These cells invade mostly the ventricles, the meninges, and can even penetrate the brain parenchyma in the affected regions.

### Toxicity of neuraminidase

The enzyme NA from *C. perfringens* used in the present investigation contains <0.1% of unspecific contaminating protease activity. Thus, we think that the effects described may be mostly attributable to the specific cleavage of sialic acid. Moreover, we did also inject NA from other sources, named the bacteria *Arthrobacter ureafaciens*, the Newcastel virus, and the influenza virus and obtained similar results. The lectin study demonstrated that the NA from *C. perfringens* cleaves sialic acid and exposes galactose residues from the ependyma and the subependymal parenchyma to a depth of about 400 μm. The desialyzation affects the injected ventricle and (at least) the rostral part of the third ventricle but not the contralateral ventricle. In previous studies using a higher dose of NA (10), desialyzation extended up to the level of the foramina in the fourth ventricle. In this location, NA seems to escape from the ventricle toward the outer CSF compartments. By this route, the NA could exert its effects in the meninges and in places located farther from the injection site such as the optic tract.

Many microbes that provoke ependymal detachment, inflammation, and hydrocephalus posses NA in their envelopes (14, 23, 24). Kelly and Greiff (11, 12) demonstrated in mice the high toxic effects of bacterial and viral NA when injected directly into the brain. Also, in humans, NA activity seems to be associated with adverse prognosis in pneumococcal meningitis (13). So much so that mump viruses with low neurovirulence due to point mutations in hemagglutinin–neuraminidase capside proteins are being used to develop new harmless vaccines (15). Thus, it seems that the sole presence of NA in the CSF may trigger inflammation that gives rise to important brain damages.

### Activation of resident cells

The activation of microglia, astrocytes, and endothelial vascular cells after the administration of NA was studied. IBA1 and GFAP, used here to identify microglia and astrocytes, respectively, are known to increase its expression after brain insults (25–27). The increase in IBA1 expression could be related to a higher mobility of microglia (28, 29). In this sense, we observed many IBA1-positive cells crossing the ependymal barrier after NA treatment and before the invasion of the brain by blood-borne cells. Interestingly, many of these cells also expressed IL1β, as observed by double immunostaining. Furthermore, we did not see any IL1β-positive cell in control specimens. This suggests that some IBA1-positive resident subependymal cells might have acquired IL1β positivity after the injection of NA, and that they move toward the lumen of the ventricle through the ependymal lining. However, since the ventricular macrophages (i.e., epiplexus cells in the choroid plexus) also expressed IBA1 and, after NA insults, begun to express IL1β, we do not have the definitive evidence to know whether the cells crossing the ependyma are indeed subependymal microglia or ventricular macrophages. IL1β is a known inflammatory mediator of the monocyte–macrophagic cell lineage (30). The capability of microglia to release IL1β was reported before in a variety of brain insults (31–33). It is likely that the increased expression of IBA1 and IL1β is related to the posterior pleocytosis, and reflects a tight relationship between the subependymal microglia and the resident ventricular macrophages. In this sense, both cell types share a common ontogenetic origin (34, 35) and may serve as sentinels of the ventricular system, probably acting as an important first-line defensive barrier to a variety of brain insults. It has been recently shown that may exists at least two subsets of microglial cells capable of increase the expression of IBA1 after experimental insults (36), one in the acute phase and the other in the chronic phase. We agree that different populations of microglial cells, as for macrophages, may play different roles in neuroinflammation after traumatic injuries. This subject deserves future investigation in our model too.

Regarding the increased expression of GFAP, the activation of astrocytes in those places more affected by the NA could be associated to the formation of a glial scar that takes place after ependymal denudation (20, 37). However, in structures located relatively far from the site of the injection, such as the stria medullaris or the optic chiasm, the activation of astrocytes and microglia is most probably related to the neuroinflammation process. It is quite evident that microglia and astrocytes play active roles in neuroinflammation but the exact mechanism of action is still controversial (38, 39).

ICAM1 is a key player in the recruitment of leukocytes during inflammation (40, 41). Its expression is increased in endothelial cells of vessels located in brain regions affected by an inflammatory insult (42–47). We also found a significant increase in the expression of ICAM1 in the cerebral blood vessels located within the regions affected by the injection of NA. In agreement to Alvarez and Teale (48), the vessels displaying a more prominent extravasation were the venules of the leptomeninges, the choroid plexus and the subependyma around the injected lateral ventricle and the foramen interventricularis. These same vessels showed the higher expression of ICAM1, which was detectable even in control non-injected specimens. Interestingly they also presented the highest expression of receptors for IL1β (49) and for other inflammatory mediators (44). This suggests that inflammatory mediators, such as IL1β, might preferentially elicited extravasation in these vessels. The functional relevance of the different permeability of parenchymal vessels versus large meningeal, choroidal, and subependymal vessels is still an open question (48, 50). On the other hand, ICAM1 was also expressed in the surface of the ependymal cells (especially in the choroid plexus) and of some ventricular cells. This could speak in favor of a role of ICAM1 in the penetration of some invading cells into the brain parenchyma (51–53).

### Infiltration of blood leukocytes

In the model here described a profuse invasion of white blood cells takes place from about 4 h after the NA-injection. The time course of such neuroinflammation was similar to that of other models in rodents (23, 54–56). The first extravasated cells were neutrophils, followed by macrophages and then lymphocytes. In agreement to Ma et al. (57), most lymphocytes were T-lymphocytes (CD3ε-positive) and of these most were CD8α-positive, as occurred in other cases of brain inflammation (58). The leukocytes crossed both the blood–brain barrier and the blood–CSF barrier, and invaded the perivascular spaces and the ventricular or meningeal CSF (59). They also reached the brain parenchyma, either directly from vessels located in the parenchyma or from the ventricle after crossing through the ventricular ependyma. The production by the invading or the resident cells of metalloproteinases that degrade specific junctional proteins seems to be essential for the invasion process (60). The passage of cells (resident or infiltrative) through the ependymal lining is still an unresolved question (61, 62) and it should also require the production of metalloproteinases (63–65). The nature of the signals involved in this trafficking of cells, the fate of the amoeboid cells, and the consequences in the periventricular tissues are still not well understood and deserve future investigations.

In summary, we describe here a novel model, compatible with life, to study the time course of aseptic meningitis and ventriculitis in rodents. The pathology, which is induced by the ICV injection of the enzyme NA, is highly reversible, and rats cure almost completely after some weeks. Apparently, only minor long lasting consequences remain in certain zones, mostly nearby the site of injection. However, these minor consequences could make the NA-treated animals more prone to develop neurological disorders, a possibility that deserves further research.

## Author Contributions

PD made the main laboratory works and hence contributed greatly to the acquisition of the information of the present paper. He drafted the work and approved the final form. They were in agreement with the contributions made by other authors. MR, JG, MM, MC, and MA supported technically all methods used in the present investigation; they also read critically the final form of the manuscript and approved it in agreement. JG also initiated the research. MA made a substantial contribution in the design and corrections of the final approved form of the manuscript, ensuring that any part of the work was appropriately investigated. Finally, FF and PL designed the research and conducted the group, making substantial contributions during all processes.

## Conflict of Interest Statement

The authors declare that the research was conducted in the absence of any commercial or financial relationships that could be construed as a potential conflict of interest.
